# Development of a ferroptosis-related signature and identification of NOTCH2 as a novel prognostic biomarker in pancreatic cancer

**DOI:** 10.3389/fimmu.2025.1659652

**Published:** 2025-10-22

**Authors:** Siyi Zhang, Xiaoxuan Li, Xiang-Xue Li, Zi-Heng Zhang, Kai-Hui Zhu, Jing Guo

**Affiliations:** 1 Department of Oncology, The Affiliated Hospital of Qingdao University, Qingdao, Shandong, China; 2 Department of Gastroenterology, Huangdao District People’s Hospital, Qingdao, Shandong, China

**Keywords:** ferroptosis, notch2, pancreatic cancer, immune infiltration, prognostic biomarker

## Abstract

**Background:**

Ferroptosis, a regulated form of iron-dependent cell death, has shown promise as an anti-tumor mechanism. However, its role in pancreatic cancer remains largely unexplored. This study aimed to identify a ferroptosis-related prognostic signature and key biomarkers.

**Methods:**

Transcriptomic profiles and clinical data of pancreatic cancer patients were obtained from the GEO and TCGA databases. A prognostic signature was constructed using LASSO and Cox regression analysis. The role of a key gene, NOTCH2, was investigated through somatic mutation, functional enrichment, immune infiltration, and drug sensitivity analysis. *In vitro*, the expression of NOTCH2 was confirmed by Western blot, and its effects on cell proliferation and migration were assessed using MTT, colony formation, and wound-healing assays. Its involvement in ferroptosis was further investigated by measuring intracellular iron, reactive oxygen species (ROS) and C11-BODIPY.

**Results:**

We constructed and validated a ferroptosis-related prognostic signature consisting of NOTCH2, KRT18, and H1-2. Patients in the high-risk group, as defined by this signature, exhibited significantly worse overall survival. A nomogram integrating the risk score and clinical variables demonstrated excellent accuracy in predicting patient prognosis. We identified NOTCH2 as a key biomarker, showing upregulated expression in pancreatic cancer tissues and cell lines, which correlated with poor prognosis and increased infiltration of M2 macrophages. Functionally, knockdown of NOTCH2 *in vitro* inhibited the proliferation and migration of pancreatic cancer cells while increasing both intracellular iron concentration and lipid peroxidation levels.

**Conclusion:**

Our study establishes a ferroptosis-related signature for prognostic prediction in pancreatic cancer and identifies NOTCH2 as a critical prognostic biomarker. NOTCH2 may promote pancreatic cancer progression by suppressing ferroptosis, highlighting it as a potential therapeutic target.

## 1 Introduction

Pancreatic cancer remains one of the most lethal malignancies, characterized by late diagnosis, rapid progression, and poor prognosis. Pancreatic ductal adenocarcinoma (PDAC) is the most common type of pancreatic cancer, accounting for more than 90% of all solid pancreatic neoplasms. According to the 2022 global cancer statistics, there were 510,566 new cases and 467,005 deaths of pancreatic cancer (1). Pancreatic cancer ranks 12th in incidence among common cancers, yet it is the 6th leading cause of cancer-related mortality. Surgery is the only potential cure for pancreatic cancer, but it is only possible in a small percentage of cases. Pancreatic cancer is often diagnosed at an advanced stage due to the lack of specific early symptoms and highly sensitive screening methods (2). Despite advances in therapeutic strategies, the five-year survival rate for pancreatic cancer patients remains dismally low (3, 4). Therefore, identifying novel biomarkers and therapeutic targets is crucial for improving the survival outcomes of pancreatic cancer patients.

Ferroptosis is an iron-dependent, lipid peroxidation-driven form of programmed cell death that plays a critical role in cancer biology (5). Morphologically, ferroptosis is primarily marked by significant mitochondrial shrinkage, increased membrane density, and the reduction or disappearance of mitochondrial cristae (6). Glutathione peroxidase 4 (GPX4) is an important antioxidant enzyme that inhibits lipid peroxidation and ferroptosis (7). GPX4 relies on reduced glutathione to eliminate lipid peroxides. Inhibition or depletion of GPX4 leads to the accumulation of lipid peroxides and triggers ferroptosis. Excessive intracellular iron levels contribute to the production of reactive oxygen species (ROS) via the Fenton reaction (8). This leads to the accumulation of lipid peroxides, particularly polyunsaturated fatty acids (PUFAs) within cell membranes (5). Enzymes such as ACSL4 and LPCAT3 facilitate the incorporation of PUFAs into cell membrane phospholipids, leading to their subsequent oxidation (9). This renders cell membranes more susceptible to ROS attack, resulting in the generation of more lipid peroxides.

Ferroptosis is associated with various physiological processes within tumor cells (10). Cancer cells more susceptible to ferroptotic cell death regulation compared to normal cells (11). Recent studies have highlighted the potential of ferroptosis as a therapeutic target in various cancers, such as hepatocellular carcinoma, breast cancer, and lung cancer (12–14). Pancreatic cancer cells, with their high metabolic demands for proliferation and DNA synthesis, are particularly dependent on intracellular iron (15). The oxidation and reduction of iron promote the production of ROS, thereby accelerating tumor growth. Therefore, serum ferritin and transferrin, which reflect iron levels, can serve as potential diagnostic biomarkers for pancreatic cancer (16). Recent research has underscored the critical role of ferroptosis in pancreatic cancer progression and treatment response. Studies have shown that high expression of NCOA4 in PDAC leads to increased NCOA4-mediated ferritinophagy, a process that supports tumor cell proliferation by maintaining iron homeostasis. Evidence from mouse models indicates that NCOA4 knockout significantly delays tumor progression and prolongs survival, whereas its overexpression accelerates tumor growth. This demonstrates that NCOA4-driven ferritinophagy is a key driver of pancreatic cancer growth and survival (17). Another study indicates that the destruction of pancreatic cells via ferroptosis triggers the release of 8-OHG, a damage-associated molecular pattern (DAMP) that signals oxidative DNA damage. This release activates the STING-dependent DNA sensor pathway, promoting macrophage infiltration and M2 polarization, which in turn facilitates pancreatic carcinogenesis (18). Furthermore, in terms of metabolic adaptability, PDAC cells resist oxidative stress by upregulating SLC7A11 and GPX4, which supports their survival in a hypoxic environment (19).

Erastin and RSL3 have been shown to induce ferroptosis and exhibit anti-tumor activity (20). Combining ferroptosis inducers with chemotherapy, radiotherapy, and immunotherapy can improve treatment outcomes by overcoming drug resistance (20, 21). However, tumor cells can also develop resistance to ferroptosis and promote cancer progression by upregulating antioxidant defenses and altering iron metabolism (22). The effect of ferroptosis on tumor depends on the release of DAMPs and the activation of immune response triggered by ferroptotic damage within the tumor microenvironment (23). A comprehensive analysis of ferroptosis-related genes (FRGs) will provide a more in-depth understanding of their impact on cancer progression. However, the role of FRGs in pancreatic cancer and their association with immune response remains largely unexplored.

In this study, we performed a comprehensive bioinformatics analysis using publicly available pancreatic cancer datasets to construct and evaluate a ferroptosis-related gene signature. This signature demonstrated robust prognostic value for overall survival (OS). Furthermore, we investigated the role of NOTCH2 in pancreatic cancer in terms of immune infiltration, prognostic significance, somatic mutation profiles, and drug sensitivity. These findings were further substantiated through experimental validation. Our findings suggest that NOTCH2 may serve as a novel biomarker and a potential therapeutic target in pancreatic cancer, offering new avenues for personalized anti-tumor strategies.

## 2 Methods

### 2.1 Data collection

RNA-sequencing, somatic mutation, and associated clinical data for pancreatic adenocarcinoma cohorts were obtained from The Cancer Genome Atlas (TCGA) database (https://portal.gdc.cancer.gov/) and the Gene Expression Omnibus (GEO) database (https://www.ncbi.nlm.nih.gov/) (24). The TCGA-PAAD cohort consists of 4 normal tissue samples and 178 tumor samples, with prognostic data available for 152 patients. The GSE15471 cohort comprises expression data from 39 tumor samples and 39 normal samples. GSE28735 and GSE85916 cohorts contain expression information and prognostic data for 84 and 79 individuals, respectively. Expression data (transcripts per million) from 167 normal pancreatic tissue samples were obtained from the GTEx database (https://www.gtexportal.org). Genes expressed in at least 50% of samples were included in the analysis. A total of 634 FRGs were retrieved from the FerrDb database (http://www.zhounan.org/ferrdb/current/). The full gene set is listed in **Supplementary Table S1**.

### 2.2 Construction of a ferroptosis-related prognostic signature

Differentially expressed genes (DEGs) between pancreatic tumor and normal tissues in the TCGA-PAAD cohort (Counts data) were identified using the “limma” package, with thresholds set at |log fold change| > 1 and false discovery rate (FDR) < 0.05. Prognosis-related genes were determined via univariate Cox regression analysis, with a P value < 0.05 considered statistically significant. FRGs that were both differentially expressed and prognostically relevant were identified using a Venn diagram. These genes were subsequently used to construct a prognostic model through least absolute shrinkage and selection operator (LASSO) regression and multivariable Cox analysis. The risk score was calculated using the following formula:


$$\text{Riskscore}=\sum_{\text{i}=1}^{\text{n}}\left(\text{Coefi }*\text{ Expi}\right)$$

Coefi is gene coefficient; Expi is gene expression; n is the number of genes in signature.

Individuals were stratified into high- and low-risk groups based on the median risk score. Kaplan-Meier survival analysis was performed to compare survival curves. The prognostic model was validated in the GSE28735 and GSE85916 cohorts. Principal component analysis (PCA) was conducted to assess the discriminative capacity of the model, while receiver-operating characteristic (ROC) curves were used to evaluate its sensitivity and specificity in predicting the prognosis of pancreatic cancer.

### 2.3 Construction and evaluation of the nomogram

In the TCGA-PAAD cohort, univariate and multivariate Cox regression analyses were performed to determine whether the risk score could serve as an independent prognostic factor. Box plots were used to evaluate the association between the risk score and clinicopathologic parameters. A nomogram integrating the risk score and clinical features was subsequently constructed, and its predictive performance was assessed using ROC and calibration curves.

### 2.4 Identification of NOTCH2 as a potential biomarker in pancreatic cancer

A pan-cancer analysis of NOTCH2 was conducted using the Xiantao Academic Tool (https://www.xiantaozi.com/), including assessments of its differential expression across various cancer types and its prognostic significance. The predictive accuracy and sensitivity of NOTCH2 for pancreatic cancer were further evaluated using ROC curves in the TCGA-PAAD and GSE15471 cohort. Patients in the TCGA-PAAD and GSE28735 datasets were stratified into high and low NOTCH2 expression groups based on median expression levels, and Kaplan-Meier analysis was conducted to assess survival differences. Somatic mutation profiles across subgroups were visualized with waterfall plots. Drug sensitivity analysis to chemotherapy and targeted therapy drugs was assessed using the “oncoPredict” package. In addition, immunohistochemical data from the Human Protein Atlas (HPA) database (https://www.proteinatlas.org/) were used to examine NOTCH2 protein expression in pancreatic cancer and normal pancreatic tissues.

### 2.5 Functional enrichment analysis

Differential gene expression analysis was performed between high and low NOTCH2 expression groups in the TCGA-PAAD cohort. Gene Ontology (GO) and Kyoto Encyclopedia of Genes and Genomes (KEGG) enrichment analyses were conducted using the “clusterProfiler” package to identify biological processes and signaling pathways associated with NOTCH2 (q-value < 0.05). In addition, gene set enrichment analysis (GSEA) was employed to further investigate the underlying mechanisms of NOTCH2 involvement. The reference molecular datasets included “c2.cp.Kegg.Hs.symbols.gmt” and “c2.cp.reactome.v2023.2.Hs.symbols.gmt”, with |NES| > 1 and q-value < 0.1 considered statistically significant.

### 2.6 Immune infiltration and immune function analysis

To assess the association between NOTCH2 expression and immune cell infiltration, we first calculated single-sample gene set enrichment analysis (ssGSEA) scores using the “GSVA” package across pan-cancer samples. The correlations between NOTCH2 expression and the infiltration levels of 28 tumor-infiltrating immune cell (TIIC) subtypes were evaluated and visualized using box plots stratified by high and low NOTCH2 expression groups. The “CIBERSORT” algorithm was then applied to evaluate the abundance and expression levels of 22 TIIC populations. Subsequently, the “ESTIMATE” algorithm was used to compute stromal, immune, and ESTIMATE scores to characterize the tumor microenvironment across pan-cancer datasets. RNA-seq data were standardized and uploaded to the Tumor Immune Dysfunction and Exclusion (TIDE) website (http://tide.dfci.harvard.edu/) to calculate TIDE, Exclusion, and Dysfunction scores for pancreatic cancer samples.

### 2.7 Cell culture and lentiviral transfection

Human pancreatic ductal epithelial cells (hTERT-HPNE) and pancreatic cancer cell lines (ASPC-1 and BXPC-3) were obtained from the Cell Bank of the Chinese Academy of Sciences. Cell line authentication was performed using short tandem repeat (STR) profiling, and all lines tested negative for mycoplasma contamination. Cells were maintained in RPMI-1640 medium supplemented with 10% fetal bovine serum (FBS) and 1% penicillin–streptomycin at 37 °C in a humidified atmosphere with 5% CO_2_. Lentiviral vectors encoding short hairpin RNA (shRNA) targeting NOTCH2 were constructed by GeneChem (Shanghai, China; http://www.genechem.com.cn/). The target lentiviral vector used was GV493, with the element sequence hU6-MCS-CBh-gcGFP-IRES-puromycin (Reference number: CON313). The RNAi negative control (sh-NC) sequence was TTCTCCGAACGTGTCACGT. The shRNA sequences targeting NOTCH2 were:

sh-NOTCH2-1, GCATGCATCAGCAATCCTTGC;
sh-NOTCH2-2, GCGGTGTACCATTGACATTGA;
sh-NOTCH2-3, GCACCTGTGAGAGGAATATTG.

ASPC-1 and BXPC-3 cells were seeded into 6-well plates at a density of 2 × 10^4^ cells per well. After 24 hours, the medium was replaced, and diluted lentiviral supernatant was added for infection. Following a 24-hour incubation, cells were transferred to culture dishes and selected with puromycin (48 hours, repeated 3 times) to establish stably transduced cell lines. Constructs exhibiting the most efficient NOTCH2 knockdown were identified and selected for subsequent experiments.

### 2.8 Quantitative real-time polymerase chain reaction

Total RNA was extracted from cells using TRIzol^®^ reagent and subsequently reverse transcribed into complementary DNA (cDNA). The qRT-PCR reaction mixture contained 2 μL of cDNA, 7.2 μL of DEPC water, 10 μL of SYBR, and 0.4 μL each of forward and reverse primers. Thermal cycling was performed under the following conditions: initial denaturation at 95 °C for 30 seconds, followed by 40 cycles of denaturation at 95 °C for 10 seconds and annealing/extension at 60 °C for 30 seconds. Melting curve analysis was performed with the following steps: 95 °C for 15 seconds, 60 °C for 60 seconds, and 95 °C for 15 seconds. Primer sequences are provided as follows:

NOTCH2-F (5′-ATGCCGGGTTTCAAAGGTGT-3′).
NOTCH-R (5′-ATGTCGATCTGGCACACTGG-3′).
β-actin F (5’-GACCACCTTCAACTCCATCAT-3’).
β-actin R (5’-CCTGCTTGCTAATCCACATCT-3’).

All experiments were performed in triplicate. β-actin was used as an internal control, and relative gene expression levels were calculated using the 2 − ΔΔCt method.

### 2.9 Western blot analysis

Cells were lysed using IP lysis buffer, and the supernatant was collected by centrifugation. Proteins (20 μg per sample) were separated by 10% SDS-PAGE and transferred onto polyvinylidene difluoride (PVDF) membranes (10600023, Cytiva, USA). Membranes were blocked with 5% skim milk for 1 hour at room temperature and then incubated overnight at 4 °C with primary antibodies (RabMab, ab307700, 1:1000, Abcam, UK). After washing three times with TBST (10 minutes per wash), membranes were incubated with secondary antibodies (M21002, Abmart, China) at 37 °C for 1 hour, followed by another three washes with TBST. Protein bands were visualized using an enhanced chemiluminescence detection system.

### 2.10 MTT assay

sh-NC and sh-NOTCH2 of ASPC-1 and BXPC-3 cell lines were seeded in 24-well plates at a density of 7 × 10³ cells per well. After 48 hours of incubation, 500 μL MTT solution (0.5 mg/mL, Aladdin, M158055) prepared with FBS-free medium was added to each well and incubated for at least 2 hours at 37 °C. Formazan crystals formed by viable cells were then dissolved in an equal volume of DMSO. Absorbance was measured at 490 nm using a microplate reader (BioTek, USA). Cell viability was calculated as follows: cell viability (%) = experimental group OD value/control group OD value×100%. Cell proliferation was assessed every 24 hours for three consecutive days. All data were processed using Microsoft Excel and visualized with GraphPad Prism 9.

### 2.11 Colony formation and wound-healing assays

For the colony formation assay, 1,000 cells were seeded into each well of a 6-well plate and cultured in a humidified incubator at 37 °C with 5% CO_2_. The culture medium was refreshed every two days. After 14 days, cell colonies were fixed at room temperature with 4% paraformaldehyde, stained with crystal violet, and imaged.

For the wound healing assay, pancreatic cancer cells were seeded into 6-well plates following transfection with sh-NC or sh-NOTCH2 lentivirus. When the cell density of the sh-NC group reached 80%-90% of the area per well, a wound was made in the cell monolayer using a 200 µL tip. After washing with phosphate-buffered saline (PBS) to remove detached cells, adherent cells were incubated in FBS-free medium. The images were obtained at 0 and 48 h. The scratch area was measured three times to evaluate the cell healing rate. The data were analyzed using ImageJ software and graphs were generated using GraphPad Prism 9 software.


$$\text{Cell healing rate}(\%)=\frac{\left(0\text{h scratch area}-48\text{h scratch area}\right)}{0\text{h scratch}}\times 100\%$$

### 2.12 Iron, ROS and C11-BODIPY detection

ASPC-1 and BXPC-3 cells were seeded into 24-well plates and cultured for 48 hours at 37 °C in a humidified incubator with 5% carbon dioxide. Cells were then incubated with FerroOrange (Dojindo, China) of 1 μmol/L for 30 minutes under identical conditions. The level of iron ion was assessed using an inverted fluorescence microscope (Nikon, Japan).

For ROS detection, H_2_DCFDA (DCF, 10 mmol/L) and dihydroethidium (DHE, 10 mmol/L) dyes were prepared in FBS-free RPMI-1640 medium and diluted to a final concentration of 10 µmol/L. Cells were pre-seeded in 24-well plates and incubated for 48 hours. Cells were incubated with the respective dye for 1 hour, washed twice with PBS. Fluorescence imaging was conducted using an inverted fluorescence microscope (Nikon, Japan).

For lipid peroxidation detection, ASPC-1 and BXPC-3 cells were seeded in small dishes and incubated for 48 hours, then 1 μM C11 BODIPY was added, and the cells were further incubated at 37 °C for 30 minutes. Subsequently, the cells were washed three times with PBS and stained with Hoechst 33342 to label cell nuclei. The level of lipid peroxidation was assessed by detecting the increase in green fluorescent signal or changes in the red-to-green fluorescent ratio. All fluorescent images were captured using a confocal microscope (Leica, Germany).

### 2.13 Statistical analysis

All statistical analyses were performed using R (version 4.4.0). Differential gene expression analysis was conducted using the “limma” package, with significance thresholds defined as |log fold change| ≥1 and P < 0.05. Box plots and volcano plots were generated using the “ggplot2” and “ggpubr” packages. Survival analysis was performed using the Kaplan-Meier method and log-rank tests, implemented through the “survival” and “survminer” packages. Nomogram was constructed with the “rms” and “regplot” packages. Mutation landscapes were visualized using the “maftools” package. Between-group comparisons were assessed using the Wilcoxon test, and correlation analyses were performed using Spearman’s correlation coefficients. One-way ANOVA and Student’s t-test were performed using GraphPad Prism (version 9.3.1). Densitometric analysis of Western blot bands, wound-healing images, and merged fluorescence images were conducted with ImageJ. A P-value < 0.05 was considered statistically significant. Significance levels were annotated as follows: ns P>0.05; *P < 0.05; **P < 0.01; ***P < 0.001.

## 3 Results

### 3.1 Identification of prognosis-related differentially expressed ferroptosis-associated genes

**Figure 1:**
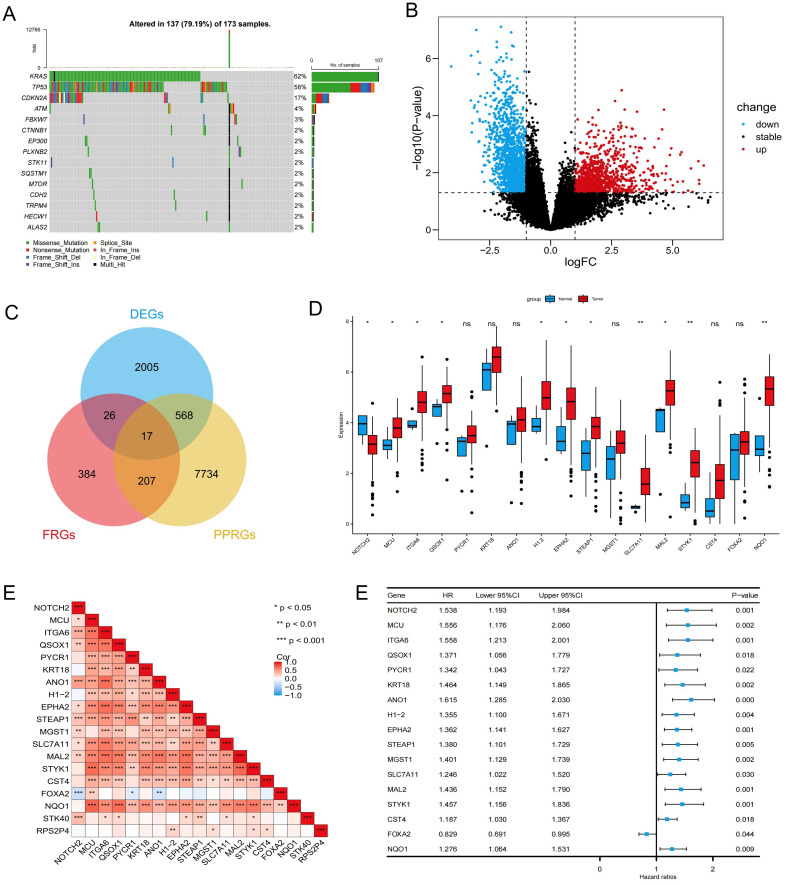
Identification and prognostic analysis of PR-DE-FRGs in pancreatic cancer. **(A)** Waterfall plot illustrating the somatic mutation frequency in the TCGA-PAAD cohort. **(B)** Volcano plot showing the differentially expressed genes in the TCGA-PAAD cohort. **(C)** Venn diagram identifying 17 PR-DE-FRGs. **(D)** Expression levels of PR-DE-FRGs in pancreatic cancer and normal samples from the TCGA database. **(E)** Spearman correlation analysis of PR-DE-FRGs. **(F)** Univariate Cox regression analysis of PR-DE-FRGs. ns P>0.05; *P < 0.05; **P < 0.01; ***P < 0.001. PR-DE-FRGs, prognosis-related differentially expressed ferroptosis-related genes; TCGA, The Cancer Genome Atlas.

Most cancer-associated mutations are somatic mutations. In the TCGA-PAAD cohort, we performed somatic mutation analysis and visualized the top 15 most frequently mutated genes using a waterfall plot (**Figure 1A**). The results revealed that KRAS exhibited the highest mutation frequency (62%), followed by TP53 and CDKN2A, with missense mutations being the predominant mutation type. Differential expression analysis identified 2,616 genes with statistically significant differences between pancreatic tumor and normal tissues (P < 0.05), including 1,003 upregulated in tumors and 1,613 upregulated in normal tissues (**Figure 1B**). Univariate Cox regression analysis further identified 8,526 pancreatic cancer prognosis-related genes (PPRGs) (p < 0.05). By intersecting the 2,616 DEGs, 634 FRGs, and the 8,526 PPRGs, we identified 17 prognosis-related differentially expressed ferroptosis-associated genes (PR-DE-FRGs) (**Figure 1C**). Boxplot analysis showed that MCU, ITGA6, QSOX1, H1-2, EPHA2, STEAP1, SLC7A11, MAL2, STYK1, and NQO1 were highly expressed in pancreatic cancer tissues, whereas NOTCH2 was predominantly expressed in normal pancreatic tissues (**Figure 1D**). The relationships among the PR-DE-FRGs were assessed using Spearman correlation analysis, which revealed a significant inverse correlation between NOTCH2 and FOXA2, and positive correlations between NOTCH2 and the majority of the other genes, including MCU, ITGA6, and SLC7A11 (**Figure 1E**). Univariate Cox regression analysis indicated that FOXA2 was the only gene associated with a favorable prognosis (hazard ratio [HR] < 1, p < 0.05), while the remaining 16 genes were significantly associated with poorer OS (HR > 1, p < 0.05) (**Figure 1F**).

### 3.2 Development and validation of a ferroptosis-related prognostic signature

**Figure 2:**
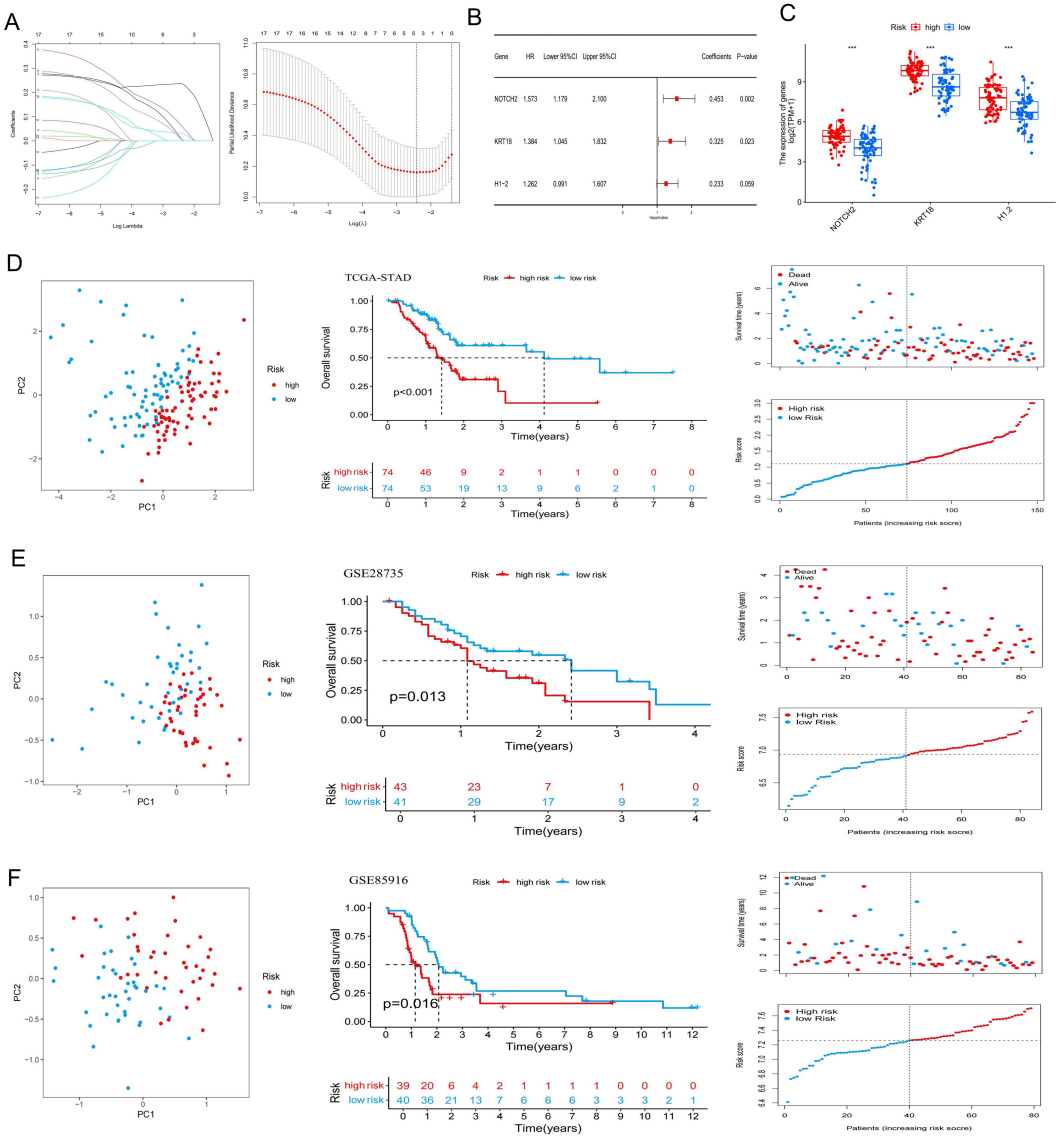
Construction of a ferroptosis-related risk signature. **(A)** Lasso Cox regression analysis and cross-validation. **(B)** Multivariate Cox analysis for determining the optimum signature genes. **(C)** Expression of the signature genes in high- and low-risk groups in the TCGA cohort. **(D–F)** Principal Component Analysis, Kaplan-Meier survival curves, and the distribution of risk scores and survival status for the high- and low-risk groups in the TCGA-PAAD, GSE28735, and GSE85916 datasets. ***P < 0.001. TCGA, The Cancer Genome Atlas.

To minimize the risk of overfitting, LASSO regression with cross-validation was applied to the 17 PR-DE-FRGs, yielding five genes (NOTCH2, KRT18, ANO1, H1-2, and MGST1) (**Figure 2A**). Subsequently, multivariate Cox regression analysis identified a three-gene prognostic signature comprising NOTCH2, KRT18, and H1-2 (**Figure 2B**). Among them, NOTCH2 exhibited the highest coefficient and the most significant association with prognosis (P<0.005). The risk score was calculated according to the following formula:

Risk score = (0.45326 × NOTCH2 expression) + (0.32491 × KRT18 expression) + (0.23260 × H1–2 expression).

**Figure 3:**
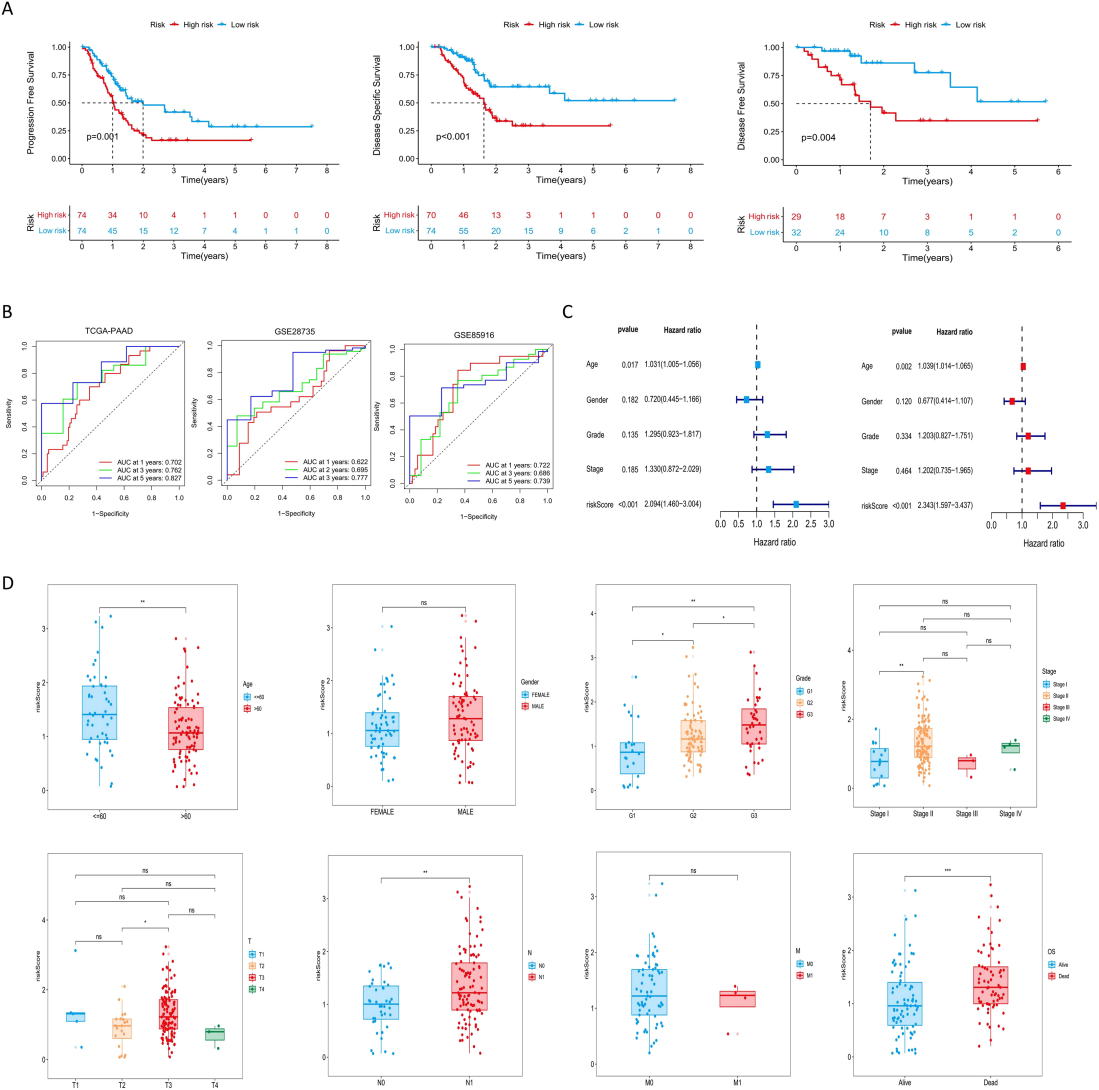
Evaluation of the risk signature and its relationship with clinicopathological parameters. **(A)** Kaplan-Meier analysis of PFS, DSS, and DFS for the risk signature in the TCGA-PAAD cohort. **(B)** ROC curves evaluating the risk signature for 1-, 3-, and 5-year survival. **(C)** Cox regression analyses of the risk score and other clinical parameters in the TCGA cohort. **(D)** Distribution of risk scores across subgroups of different clinicopathological parameters. ns P>0.05; *P < 0.05; **P < 0.01; ***P < 0.001. PFS, progression-free survival; DSS, disease-specific survival; DFS, disease-free survival; TCGA, The Cancer Genome Atlas; ROC, Receiver Operating Characteristic.

In the TCGA-PAAD cohort, patients were stratified into high-risk and low-risk groups based on the median risk score (**Supplementary Table S2**). As shown in **Figure 2C**, expression levels of NOTCH2, KRT18, and H1–2 were significantly elevated in the high-risk group (P<0.001). PCA demonstrated clear separation between the two risk groups, indicating the model’s discriminatory capacity. Kaplan-Meier analysis revealed a significantly longer OS in the low-risk group (P < 0.001), and survival outcome analyses further confirmed that higher risk scores were associated with increased mortality risk (**Figure 2D**). Similar findings were observed in the GSE28735 and GSE85916 validation cohort (**Figures 2E, F**). Moreover, in the TCGA-PAAD cohort, patients in the low-risk group exhibited significantly improved progression-free survival (PFS), disease-specific survival (DSS), and disease-free survival (DFS), as compared with those in the high-risk group (**Figure 3A**). Time-dependent ROC curves demonstrated the predictive accuracy of this signature. The areas under the curve (AUC) for 1-year, 3-year, and 5-year overall survival (OS) were 0.702, 0.762, and 0.827, respectively, indicating its high sensitivity and specificity. This signature was further validated in the GSE28735 and GSE85916 cohorts (**Figure 3B**). Univariable Cox regression analysis incorporating clinical variables revealed that age (HR = 1.031, 95% CI: 1.005-1.056, P = 0.017) and risk score (HR = 2.094, 95% CI: 1.460-3.004, P < 0.001) was significantly associated with OS. Multivariable analysis confirmed age (HR = 1.039, 95% CI: 1.014-1.065, P = 0.002) and risk score (HR = 2.343, 95% CI: 1.597-3.437, P < 0.001) as independent prognostic factors (**Figure 3C**). Further subgroup analyses showed that risk scores were significantly elevated among patients who were 60 years of age or younger, had high-grade tumors (G3), lymph node metastasis (N1), or had died. No statistically significant differences in risk score were observed across sex, tumor stage, T stage, or M stage subgroups (**Figure 3D**).

### 3.3 Construction of a risk score-related prognostic nomogram

**Figure 4:**
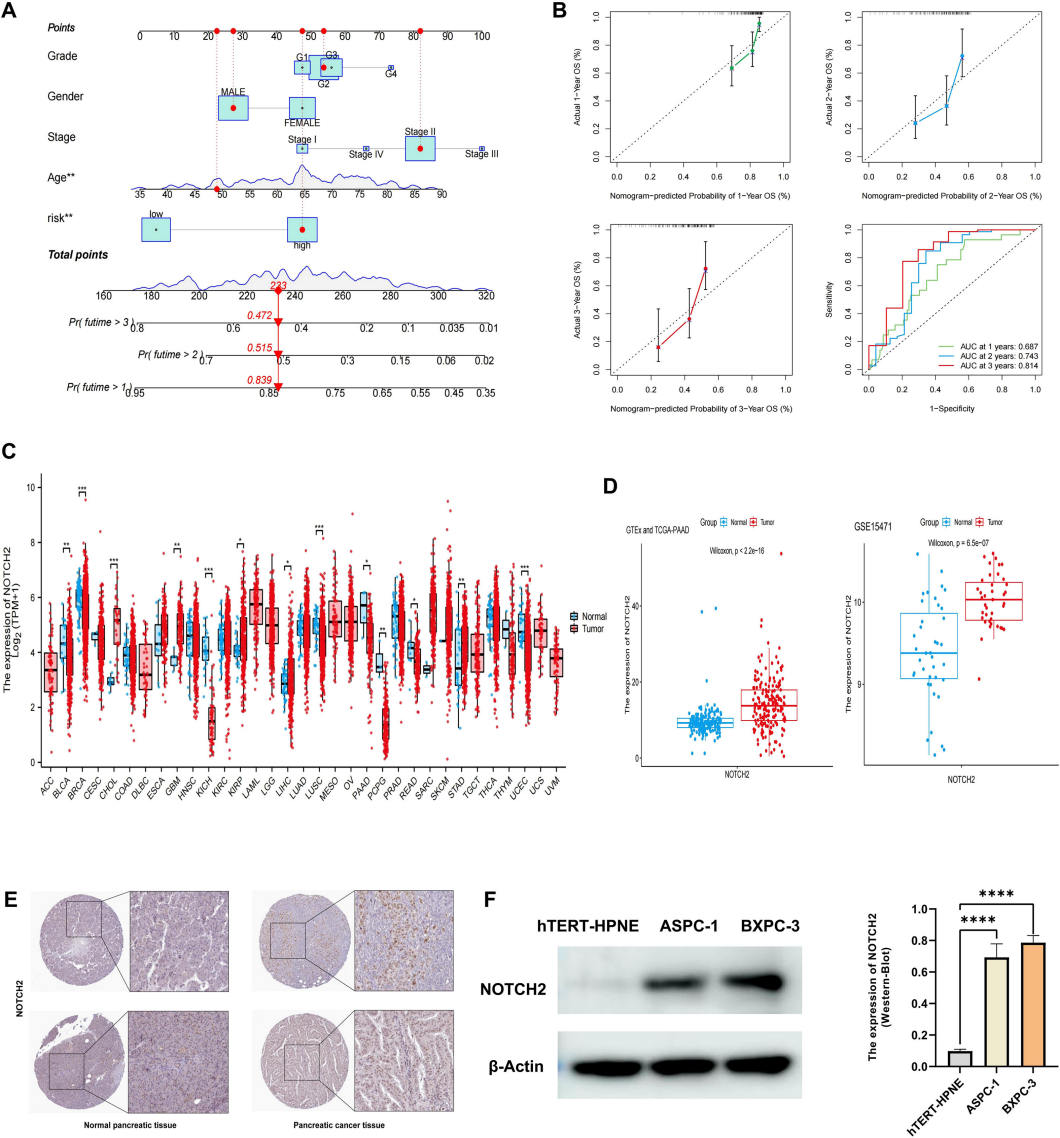
Construction of nomogram and validation of NOTCH2 expression. **(A)** A nomogram constructed based on the risk score and other clinicopathological parameters. **(B)** Calibration curves and ROC curves for the nomogram’s prediction of 1-, 2-, and 3-year survival. **(C)** Pan-cancer expression of NOTCH2 in unpaired samples from the TCGA database. **(D)** NOTCH2 expression levels in GTEx-TCGA cohort and the GSE15471 cohort. **(E)** Immunohistochemistry staining for NOTCH2 from the HPA database. **(F)** Western blot analysis of NOTCH2 expression levels in the hTERT-HPNE, ASPC-1, and BXPC-3 cell lines. ns P>0.05; *P < 0.05; **P < 0.01; ***P < 0.001. ROC, Receiver Operating Characteristic; TCGA, The Cancer Genome Atlas; HPA, Human Protein Atlas.

A prognostic nomogram was developed by integrating the risk score with clinicopathologic factors, including age, sex, tumor stage, and grade, to enhance the accuracy of survival prediction in patients with pancreatic cancer (**Figure 4A**). The AUC of 1-, 2-, and 3-year survival was 0.687, 0.743, and 0.814, respectively. Calibration curves demonstrated strong concordance between predicted and observed survival probabilities, indicating high predictive performance of the nomogram (**Figure 4B**). These findings suggest that the nomogram provides a potential tool for individualized survival prediction in pancreatic cancer patients.

### 3.4 Identification of NOTCH2 as a potential biomarker in pancreatic cancer

Among the prognostic signature genes, NOTCH2 was selected for further investigation based on its highest regression coefficient and most significant P-value. Pan-cancer analysis of the TCGA dataset revealed elevated NOTCH2 expression in several tumor types, including CHOL, GBM, KIRP, and STAD, whereas its expression was markedly reduced in BLCA, KICH, and PAAD (**Figure 4C**). However, given that the TCGA-PAAD dataset includes only four normal pancreatic tissue samples, potential sampling bias may affect these findings. To address this limitation, we integrated transcriptomic data from the TCGA and GTEx databases to compare NOTCH2 expression between pancreatic tumor and normal tissue. This analysis demonstrated significantly higher expression of NOTCH2 in pancreatic cancer. Consistent findings were observed in the GSE15471 cohort (**Figure 4D**). Immunohistochemical staining from the HPA database confirmed higher NOTCH2 protein expression in pancreatic cancer tissue, with predominant nuclear localization (**Figure 4E**). This observation was further supported by Western blot analysis, which showed significantly increased NOTCH2 protein levels in ASPC-1 and BXPC-3 cell lines (**Figure 4F**).

**Figure 5:**
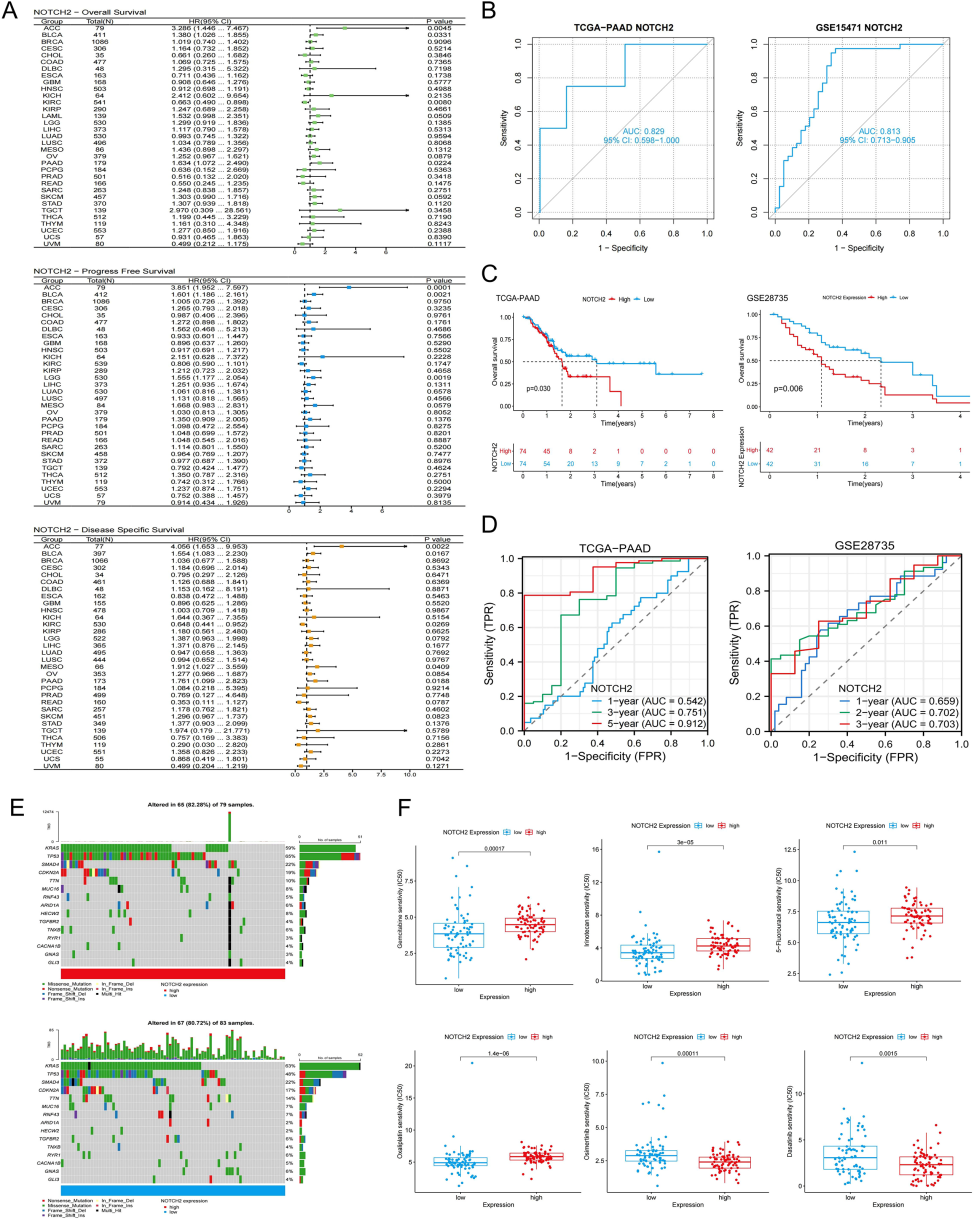
Prognostic, somatic mutation, and drug sensitivity analysis of NOTCH2. **(A)** Pan-cancer Cox regression analysis of NOTCH2 on OS, PFS, and DSS in the TCGA cohort. **(B)** Diagnostic ROC curves for NOTCH2 in the TCGA-PAAD and GSE15471 cohorts. **(C)** Kaplan-Meier analysis of OS in patients with high and low NOTCH2 expression in the TCGA-PAAD and GSE28735 cohort. **(D)** ROC curves evaluating the NOTCH2 for predicting survival in the TCGA-PAAD and GSE28735 cohorts. **(E)** Waterfall plots illustrating somatic mutations in the high- and low-NOTCH2 expression groups. **(F)** Drug sensitivity analysis of chemotherapy drugs between high- and low-NOTCH2 expression groups. OS, overall survival; PFS, progression free survival; DSS, disease specific survival; TCGA, The Cancer Genome Atlas; ROC, Receiver Operating Characteristic.

To evaluate the prognostic significance of NOTCH2 expression across tumor types, univariate Cox regression analysis were performed in the TCGA pan-cancer cohort. NOTCH2 expression was found to be significantly associated with poor prognosis in several cancers. In analyses of OS and DSS, elevated NOTCH2 expression was identified as a risk factor in ACC, BLCA, and PAAD (HR>1, P<0.05). Conversely, in KIRC, NOTCH2 appeared to serve as a protective factor (HR<1, P<0.05). ROC analysis demonstrated that NOTCH2 had strong diagnostic performance for pancreatic cancer, with an AUC of 0.829 in the TCGA-PAAD cohort and 0.823 in the GSE15471 cohort (**Figure 5B**). Pancreatic cancer patients were stratified into high- and low-NOTCH2 expression groups based on the median expression level. Kaplan-Meier analysis demonstrated that patients in the high-NOTCH2 group had significantly shorter OS (**Figure 5C**). Notably, NOTCH2 also demonstrated excellent prognostic accuracy for 5-year survival, with an AUC>0.9 in the TCGA-PAAD cohort, which was validated in the GSE28735 cohort (**Figure 5D**). Somatic mutation analysis of NOTCH2 subgroups showed a higher overall mutation frequency in the high-NOTCH2 group (82.28% vs. 80.72%). TP53 was the most frequently mutated gene in the high-NOTCH2 group (65%), whereas KRAS mutations predominated in the low-NOTCH2 group (63%) (**Figure 5E**). These observations suggest that NOTCH2 expression may be linked to distinct patterns of driver mutations, potentially contributing to variations in clinical outcomes.

### 3.5 Drug sensitivity analysis

We assessed the sensitivity of 198 therapeutic agents based on data derived from the Genomics of Drug Sensitivity in Cancer (GDSC) database. The results suggested that the expression level of NOTCH2 may influence the responsiveness to several commonly used chemotherapeutic and targeted agents. As shown in **Figure 5F**, patients with high NOTCH2 expression exhibited elevated IC_50_ values for Gemcitabine, 5-fluorouracil, irinotecan, and oxaliplatin, indicating potential resistance to these agents. Conversely, lower IC_50_ values were observed for osimertinib and dasatinib, suggesting that patients with high NOTCH2 expression may derive greater benefit from these treatments.

### 3.6 Functional enrichment analysis

**Figure 6:**
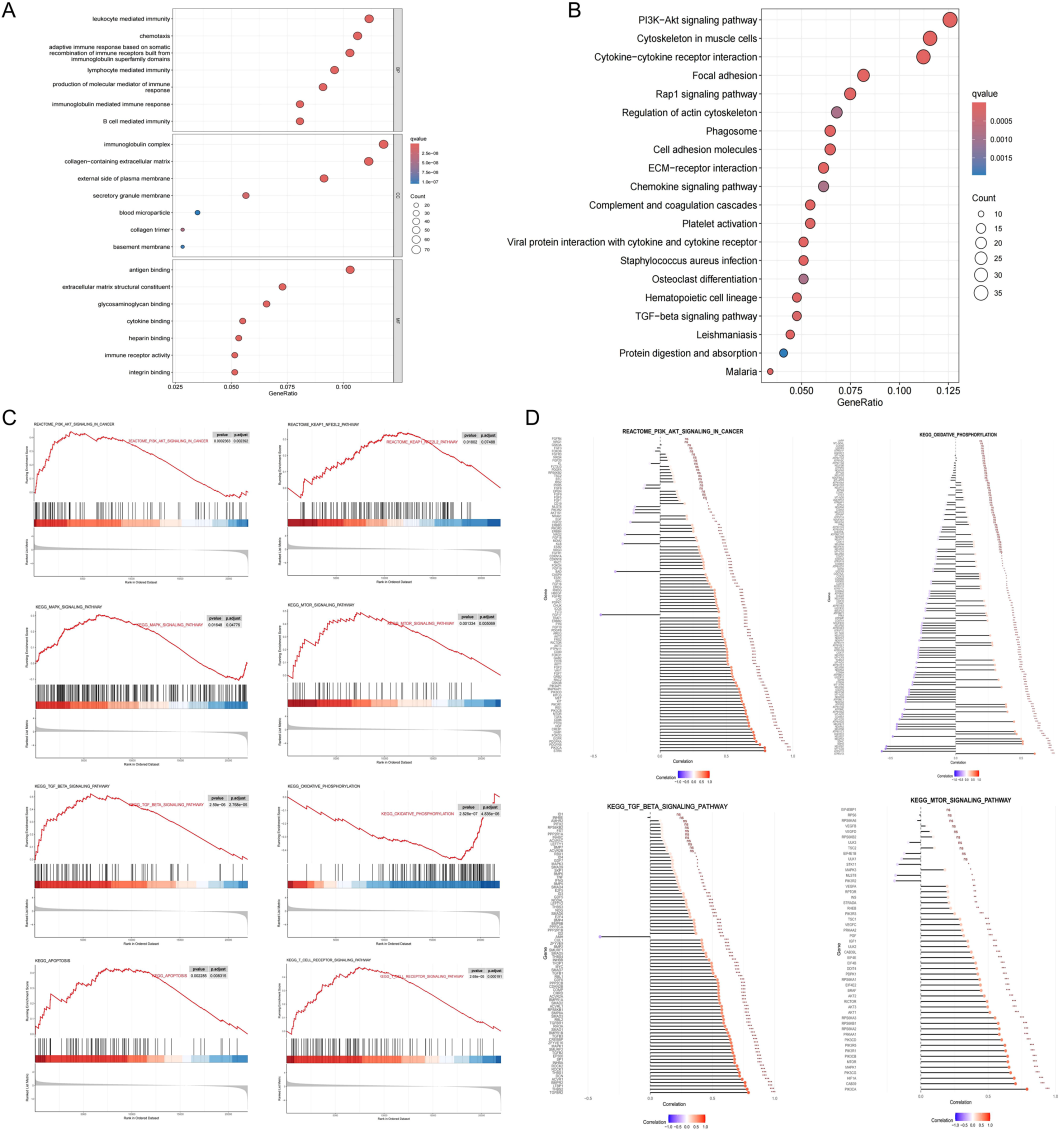
Functional enrichment analysis. GO **(A)** and KEGG **(B)** pathway enrichment analysis of DEGs between the high- and low-NOTCH2 expression groups. **(C)** GSEA analysis for the high- and low-NOTCH2 expression groups based on the KEGG and Reactome gene sets. **(D)** The Correlation between NOTCH2 and the Expression of Genes in Multiple Signaling Pathways. GO, Gene Ontology; KEGG, Kyoto Encyclopedia of Genes and Genomes; DEGs, differentially expressed genes; GSEA, gene set enrichment analysis.

Differential expression analysis between the high and low NOTCH2 expression groups identified a total of 737 DEGs, comprising 679 upregulated and 58 downregulated genes (|log fold change| > 1, FDR < 0.05). To explore the biological relevance of these genes, functional enrichment analysis were performed. As illustrated in **Figure 6A**, GO enrichment revealed that, at the Biological Process (BP) level, these DEGs were primarily involved in “leukocyte mediated immunity,” “lymphocyte mediated immunity,” and “B cell mediated immunity.” At the Cellular Component (CC) level, significant enrichment was observed in the “immunoglobulin complex” and the “collagen-containing extracellular matrix.” In terms of Molecular Function (MF), DEGs were enriched in pathways such as “antigen binding,” “cytokine binding,” and “immune receptor activity.” Collectively, these findings suggest that NOTCH2 may play a role in immune responses and antigen-antibody interactions, thereby contributing to the regulation of host immune surveillance. Moreover, KEGG pathway enrichment analysis revealed that the DEGs were significantly associated with key oncogenic signaling pathways, including the PI3K-Akt, Rap1, and transforming growth factor β (TGF-β) pathways, as well as with cell adhesion (**Figure 6B**), all of which are known to be critical in tumor initiation and metastasis (**Supplementary Table S3**). To further investigate the functional differences between high and low NOTCH2 expression groups, GSEA was performed based on KEGG and Reactome datasets. The high-NOTCH2 expression group showed significant enrichment in multiple tumor-promoting pathways, including PI3K-AKT, TGF-β, MAPK, mTOR, and KEAP1-NFE2L2 signaling. In contrast, tumors with low NOTCH2 expression were primarily enriched in mitochondrial metabolic pathways, such as oxidative phosphorylation (**Figure 6C**). To further explore the regulatory role of NOTCH2, we generated bar charts to analyze the correlation between NOTCH2 and genes in these pathways. Among them, NOTCH2 exhibits a significant positive correlation with most genes in the PI3K-AKT, TGF-β, and mTOR pathways; in particular, the correlation coefficients between NOTCH2 and genes such as PIK3CA, TGFBR2, STRN, and HIF1A exceed 0.75, with statistically significant differences. These genes may be key upstream or downstream genes of NOTCH2. These findings suggest that elevated expression of NOTCH2 may promote tumor progression and metastasis by activating oncogenic signaling, ultimately contributing to adverse clinical outcomes.

### 3.7 Assessment of immune cell infiltration and immune function

**Figure 7:**
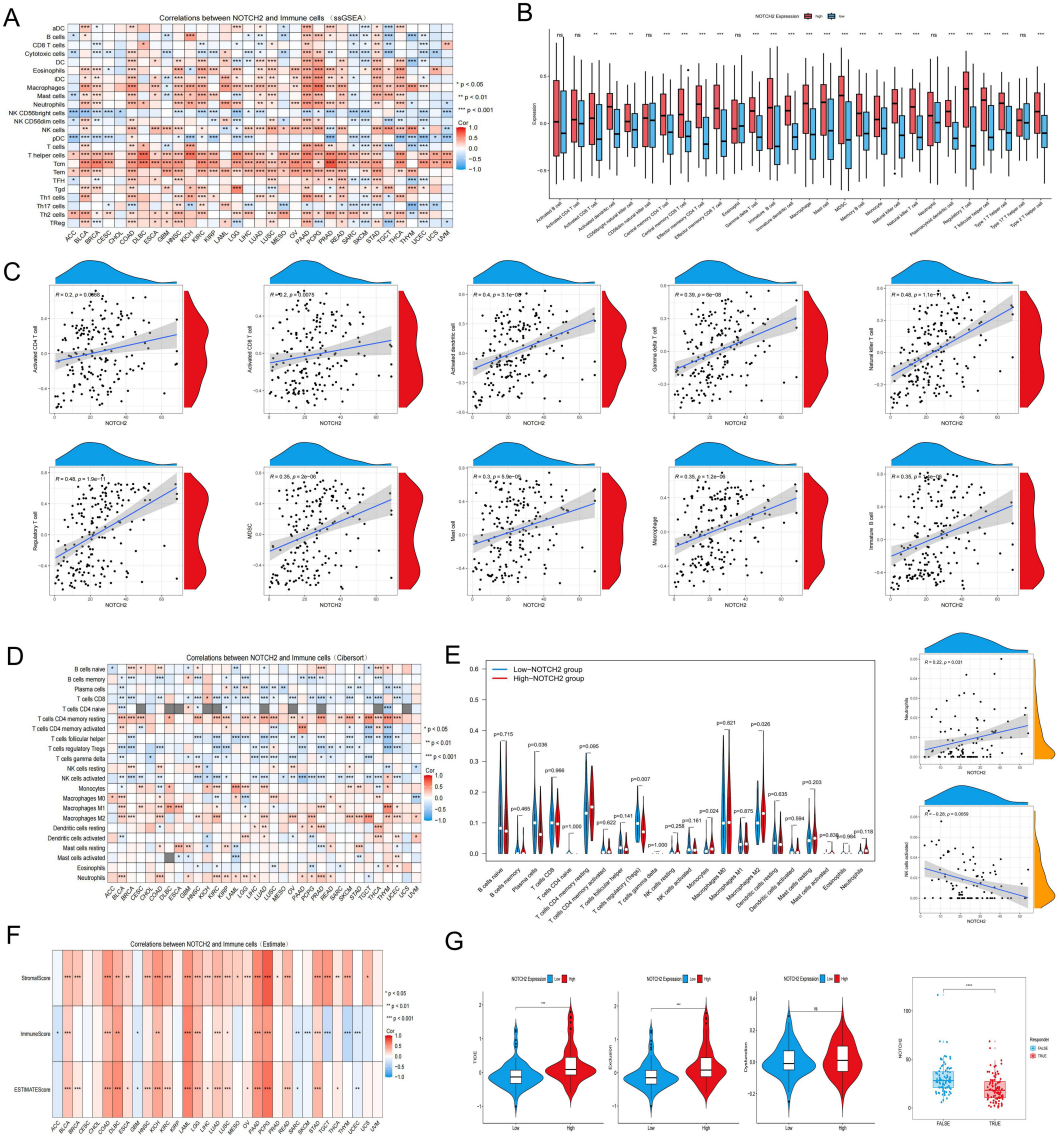
Immune cell infiltration analysis. **(A)** Correlation analysis between NOTCH2 expression and immune cells using the ssGSEA algorithm in the TCGA pan-cancer cohort. **(B)** Infiltration levels of TIICs in the high- and low-NOTCH2 expression group in the TCGA-PAAD cohort. **(C)** Correlation analysis between NOTCH2 expression and TIICs. **(D)** The relationship between NOTCH2 expression and immune cells using the CIBERSORT algorithm in the TCGA pan-cancer cohort. **(E)** The infiltration and correlation of TIICs and NOTCH2 expression using the CIBERSORT algorithm. **(F)** Correlation between NOTCH2 expression and the Stromal Score, Immune Score, and ESTIMATE Score using the ESTIMATE algorithm in the TCGA pan-cancer cohort. **(G)** The tumor immune dysfunction, exclusion score, and predicted immunotherapy response of different NOTCH2 expression groups. ns P>0.05; *P < 0.05; **P < 0.01; ***P < 0.001. ssGSEA, single-sample gene set enrichment analysis; TCGA, The Cancer Genome Atlas; TIICs, tumor-infiltrating immune cells.

TIICs represent critical components of the tumor microenvironment and play critical role in regulating tumor initiation, progression, and immune evasion. To examine the relationship between NOTCH2 expression and immune infiltration, we employed a comprehensive pan-cancer analysis using the ESTIMATE, CIBERSORT, and ssGSEA algorithms. ssGSEA revealed that NOTCH2 expression was significantly correlated with immune cell infiltration across multiple tumor types, with particularly strong associations observed in COAD, LUSC, and PAAD (**Figure 7A**). In the TCGA-PAAD cohort, high NOTCH2 expression was associated with increased infiltration of several immune cells, including macrophages, mast cells, myeloid-derived suppressor cells (MDSCs), monocytes, and regulatory T cells (Tregs) (**Figure 7B**). Spearman correlation analysis further demonstrated a significant positive association between NOTCH2 expression and the degree of immune cell infiltration mentioned above. (**Figure 7C**). Subsequently, the CIBERSORT algorithm was employed to quantify the relative abundance of 22 TIICs in each tumor sample. Compared with the low-NOTCH2 group, patients with high NOTCH2 expression exhibited a significantly higher proportion of M2 macrophages, a subtype associated with immunosuppressive phenotypes and malignant tumor progression. In contrast, increased plasma cell infiltration was observed in the low-NOTCH2 group, suggesting a potential role in anti-tumor immunity. Spearman correlation analysis revealed a significant positive association between NOTCH2 expression and neutrophil infiltration, and an inverse correlation with activated NK cells (**Figures 7D, E**). Similar findings were obtained using the ESTIMATE algorithm, which demonstrated that NOTCH2 expression was positively correlated with StromalScore, ImmuneScore, and ESTIMATEScore across multiple cancer types, including pancreatic cancer, implying a role for NOTCH2 in modulating the tumor immune microenvironment (**Figure 7F**). We further explored the potential influence of NOTCH2 on tumor immune evasion. As shown in **Figure 7G**, patients in the high NOTCH2 group had elevated TIDE scores and Dysfunction Scores. Moreover, NOTCH2 expression levels were significantly lower in immunotherapy responders compared to non-responders. These findings suggest that high NOTCH2 expression may contribute to immune escape by fostering an immunosuppressive tumor microenvironment.

### 3.8 Functional validation of NOTCH2 in pancreatic cancer

**Figure 8:**
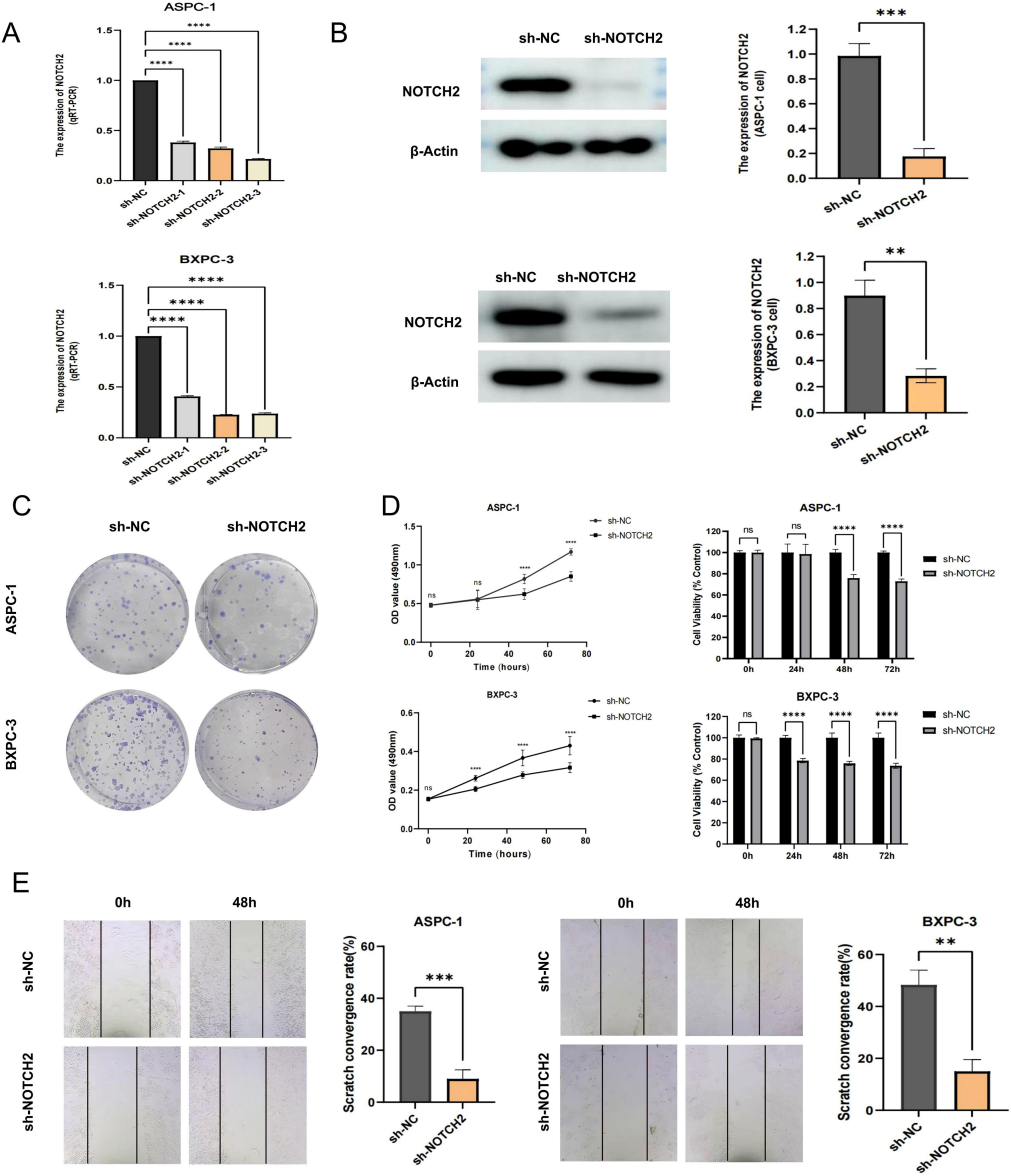
Functional validation of NOTCH2 in pancreatic cancer cells. **(A)** The expression of NOTCH2 in control (sh-NC) and knockdown (sh-NOTCH2) group of ASPC-1 and BXPC-3 cell was examined using qRT-PCR. **(B)** Western blot analysis confirmed the expression level of NOTCH2 in the sh-NC and sh-NOTCH2 group. **(C, D)** The colony formation and MTT assays in and sh-NC and sh-NOTCH2 group of ASPC-1 and BXPC-3 cell lines. **(E)** The wound-healing experiment assessed the migration capacity of ASPC-1 and BXPC-3 cells. ns P > 0.05; *P < 0.05; **P < 0.01; ***P < 0.001. **** P < 0.0001. qRT-PCR, quantitative real-time polymerase chain reaction.

To further elucidate the role of NOTCH2 in the progression of pancreatic cancer, we performed a series of *in vitro* functional assays. Lentiviral vectors carrying short hairpin RNAs targeting NOTCH2 (sh-NOTCH2) and negative control vectors (sh-NC) were constructed and transduced into ASPC-1 and BXPC-3 cell lines. Knockdown efficiency was confirmed by qRT-PCR (**Figure 8A**, **Supplementary Table S4**) and Western blotting (**Figure 8B**, **Supplementary Table S5**). Among the constructs, sh-NOTCH2–2 in ASPC-1 and sh-NOTCH2–3 in BXPC-3 demonstrated the most pronounced knockdown efficiency and were thus selected for subsequent experiments.

In MTT and colony formation assays, NOTCH2 knockdown markedly reduced cell viability and clonogenic potential in both cell lines (**Figures 8C, D**). Furthermore, in 48-hour wound-healing assays, cells in the sh-NOTCH2 group exhibited significantly impaired migratory capacity compared to controls (**Figure 8E**). Collectively, these findings indicate that knockdown of NOTCH2 suppresses the proliferation and migration of pancreatic cancer cells.

**Figure 9:**
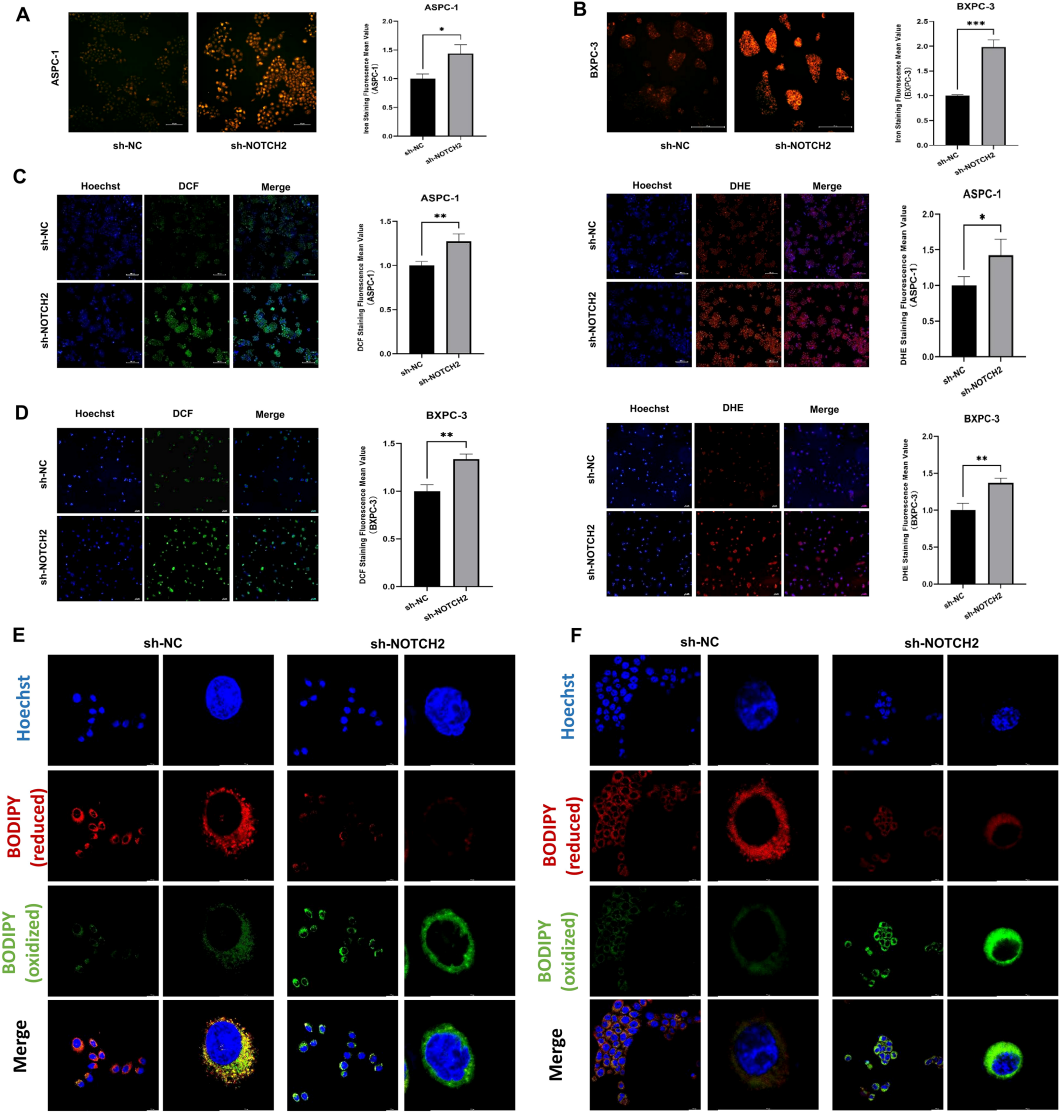
Iron staining and oxidative stress analysis. **(A, B)** Iron staining was used to detect intracellular iron levels from the sh-NC and sh-NOTCH2 groups. **(C, D)** Staining for ROS was performed to assess oxidative stress levels from the sh-NC and sh-NOTCH2 groups. **(D, E)** C11 BODIPY staining is used to assess cellular lipid peroxidation levels. ROS, reactive oxygen species; *P < 0.05; **P < 0.01; ***P < 0.001.

To investigate whether NOTCH2 is involved in the regulation of ferroptosis and oxidative stress, we performed intracellular iron detection, ROS assay, and C11 BODIPY staining. The fluorescent probe FerroOrange was used to detect intracellular Fe^2+^ levels. In both ASPC-1 and BXPC-3 cell lines, cells transduced with sh-NOTCH2 exhibited a significant increase in FerroOrange fluorescence intensity, indicating elevated intracellular free iron levels (**Figures 9A, B**). To evaluate ROS accumulation, we used the fluorescent probes DCFH-DA and DHE to measure the levels of total ROS and superoxide anions, respectively. The sh-NOTCH2 group showed significantly higher fluorescence intensity, suggesting increased oxidative stress (**Figures 9C, D**, **Supplementary Table S6**). Furthermore, the results of C11 BODIPY staining revealed that the intracellular lipid peroxidation level was significantly increased in the sh-NOTCH2 group (**Figures 9E, F**). These findings indicate that NOTCH2 knockdown leads to significant increases in intracellular iron accumulation, ROS levels, and lipid peroxidation. Therefore, NOTCH2 is a potential regulator of ferroptosis and redox homeostasis in pancreatic cancer.

## 4 Discussion

Pancreatic cancer is characterized by aggressive biological behavior and high mortality, and exhibits limited responsiveness to conventional chemoradiotherapy. Most patients with pancreatic cancer present with advanced-stage disease at diagnosis, rendering them ineligible for curative surgical resection (25). Although immune checkpoint inhibitors (ICIs) have shown considerable efficacy across various solid tumors in recent years, their effectiveness in pancreatic cancer remains poor, which may be due to the immunosuppressive tumor microenvironment (26). Emerging evidence suggests that the induction of ferroptosis can suppress pancreatic tumor growth and metastasis (27, 28). Moreover, vaccination with early-stage ferroptotic cancer cells has been shown to elicit durable antitumor immune responses (29). Accordingly, elucidating the regulatory mechanisms of ferroptosis and identifying reliable ferroptosis-related prognostic biomarkers in pancreatic cancer are of critical importance for improving patient outcomes.

This study focused on ferroptosis and established a ferroptosis-related prognostic signature. A corresponding risk score was developed, which effectively stratified patients with pancreatic cancer into high- and low-risk subgroups. Patients in the high-risk group exhibited significantly reduced OS. Notably, the risk score was identified as an independent prognostic factor. By integrating the risk score with key clinical variables, we constructed a nomogram that demonstrated good predictive performance, offering a novel approach for prognostic assessment and individualized management in pancreatic cancer.

In this study, NOTCH2 was identified as a potential biomarker for pancreatic cancer. As a key member of the NOTCH receptor family, NOTCH2 signaling is initiated by ligand binding (e.g., Jagged or Delta-like), followed by sequential proteolytic cleavages mediated by ADAM metalloproteinases and the γ-secretase complex (30). This process releases the NOTCH2 intracellular domain (N2ICD), which translocates into the nucleus, associates with the transcription factor CSL (also known as RBP-Jκ), and activates downstream target genes such as HES and HEY (31), thereby regulating proliferation, differentiation, apoptosis, and stem cell maintenance (32). Dysregulation of the NOTCH signaling is implicated in the pathogenesis of many cancers, yet the specific role of NOTCH2 in solid tumors remains controversial. In gastric cancer, some studies have reported that strong cytoplasmic or nuclear NOTCH2 expression correlates with favorable prognosis, suggesting a tumor-suppressive role (33). Conversely, other evidence indicates that high NOTCH2 expression is associated with increased gastric cancer risk, advanced TNM stage, and poor outcomes (34). Mechanistically, it has been shown that NUSAP1 can stabilize NOTCH2 by inhibiting its ubiquitination, thereby activating NOTCH2 signaling and promoting tumor progression and chemoresistance (35). Similarly, in a mouse model of esophageal disease, overexpression of activated NOTCH2 impaired goblet cell maturation, increased crypt fission, and accelerated tumor development at the squamocolumnar junction (36). By contrast, in esophageal squamous cell carcinoma, NOTCH signaling promoted keratinocyte differentiation and exerted an anti-cancer effect (37).

During the development of pancreatic cancer, NOTCH2 exhibits a dynamic expression pattern and contradictory roles. In a KRAS mutation-driven mouse model, NOTCH2 knockout prevented the progression of pancreatic intraepithelial neoplasia (PanIN) and prolonged survival. However, it also led to a phenotypic transition toward a more invasive and undifferentiated form of PDAC, a process that was closely associated with EMT. In PDAC cells, the Midkine-NOTCH2 interaction activated NOTCH signaling, induced EMT, and upregulated NF-κB, thereby promoting invasion and metastasis (38). Moreover, NOTCH2 overexpression was significantly associated with chemoresistance (39), and analysis of clinical samples demonstrated that NOTCH2 protein expression was elevated in PDAC tissues and positively correlated with metastatic tendency (40). Mechanistically, NOTCH2 exerted its oncogenic functions through the activation of a cascade of downstream targets. 1.MYC signaling: NOTCH2 acted as a direct upstream regulator of MYC transcription. During the early stages of pancreatic cancer, activation of NOTCH2 signaling led to abnormal MYC upregulation, which drove proliferation and malignant transformation of precancerous lesions (41). 2.HES/HEY transcriptional repressors: As classical downstream targets of the NOTCH pathway, HES1 and HEY1 played pivotal roles in mediating the functions of NOTCH2. HES1 expression was significantly upregulated in pancreatic cancer tissues and correlated with poor prognosis (42), while HEY1 was also identified as a prognostic biomarker (43). By regulating genes involved in cell cycle progression and differentiation, these transcriptional repressors supported tumor cell survival and proliferation. 3.EMT and tumor invasion-metastasis: NOTCH2 signaling acted as a potent inducer of EMT by directly upregulating core EMT transcription factors such as Slug and Snail-1 (39). Inhibition of NOTCH2 or its ligands reversed the EMT phenotype and attenuated the invasive potential of tumor cells (44). Taken together, these findings suggested that during the PanIN stage, NOTCH2 primarily exerted tumor-suppressive functions by maintaining differentiation and suppressing aberrant proliferation. In contrast, at the PDAC stage, NOTCH2 overexpression mediated oncogenic effects by regulating MYC signaling, driving EMT, and promoting chemoresistance. These functional transitions appeared to be tightly modulated by microenvironmental factors, epigenetic regulation, and signaling pathway crosstalk.

GEM is a cornerstone chemotherapeutic agent for pancreatic cancer treatment, yet acquired resistance remains a leading cause of treatment failure. The Notch signaling pathway has consistently been identified as a critical mediator of this resistance, with multiple studies explicitly linking NOTCH2 to this resistant phenotype. In pancreatic cancer cells with *in vitro*-induced GEM resistance (GR cells), NOTCH2 and its ligand Jagged-1 are significantly upregulated. This upregulation is accompanied by epithelial-mesenchymal transition (EMT), a process known to be a key driver of chemoresistance. The occurrence of EMT is evidenced by the downregulation of the epithelial marker E-cadherin and the upregulation of the mesenchymal marker Vimentin. Notably, either pharmacological or genetic inhibition of the NOTCH signaling pathway can partially reverse this EMT phenotype and restore sensitivity to GEM (44, 45). Furthermore, pancreatic stellate cells (PSCs) within the TME have been found to promote GEM resistance in pancreatic cancer cells by activating the NOTCH signaling pathway, specifically involving Jagged-1 and its downstream target gene Hes1 (42).

This study demonstrated the upregulated expression of NOTCH2 in pancreatic cancer tissues and its correlation with adverse clinical outcomes. *In vitro* functional assays confirmed that knockdown of NOTCH2 inhibited the proliferation and migration of pancreatic cancer cells. Functional enrichment analysis further supported the oncogenic role of NOTCH2, revealing significant associations between its high expression and hallmark signaling pathways implicated in cancer progression, including the PI3K-AKT, TGF-β, MAPK, mTOR, and KEAP1-NFE2L2 signaling. The PI3K/AKT pathway is a pivotal signaling cascade that governs cell growth, survival, and metabolism, and it is frequently hyperactivated in pancreatic cancer. Extensive evidence demonstrates that extensive crosstalk exists between the NOTCH signaling pathway and the PI3K/AKT pathway (46). Specifically, activation of the NOTCH signaling pathway in pancreatic cancer cells modulates the expression level of p-AKT, thereby influencing cell growth and migration (47). This finding strongly suggests that NOTCH2 may exert its biological effects by modulating the PI3K/AKT axis. High-throughput approaches such as chromatin immunoprecipitation sequencing (ChIP-seq) have demonstrated that NOTCH1 and NOTCH2 exhibit distinct chromatin binding profiles in pancreatic cancer cell lines (e.g., BXPC3), suggesting functional divergence and target gene specificity. Enrichment analysis indicates that NOTCH2 target genes are involved in multiple critical pathways, including PI3K-AKT, Ras, MAPK, and metabolism-related signaling. In addition to MYC, several other candidate targets such as CDKN1A and MET have also been identified (48). However, further experimental studies are still required to confirm the direct regulatory role of NOTCH2 in pancreatic cancer. In addition, we observed a potential association between NOTCH2 expression levels and the mutational status of key oncogenic drivers. TP53 mutations were more frequently detected in tumors with high NOTCH2 expression, whereas KRAS mutations predominated in tumors with low NOTCH2 expression. These findings suggest that NOTCH2 may play a role in the progression of distinct molecular subtypes of pancreatic cancer, or that its expression may be modulated by differing genomic contexts. Such observations may offer new insight into the molecular heterogeneity of pancreatic cancer.

Furthermore, KRT18 and H1–2 were included in the prognostic signature we constructed; however, these two are not common core regulators of ferroptosis. KRT18, a type I intermediate filament protein, is essential for maintaining cell cytoskeleton and has been reported to be upregulated in several malignancies, including gastric, colorectal, and liver cancers (49). Its oncogenic role is thought to be mediated through PI3K/AKT, Wnt, and MAPK/ERK pathways (50). Notably, experimental evidence in rat models of hypobaric hypoxia suggests that KRT18 may also regulate both apoptosis and ferroptosis through the JNK pathway, highlighting a possible mechanistic link (51). H1-2, a member of the histone H1 family, is primarily known for its role in maintaining genomic stability and participating in the DNA damage response. Under certain conditions, H1–2 can translocate from the nucleus to mitochondria and contribute to apoptosis regulation (52). However, to date, no published studies have reported the regulatory mechanism of H1–2 on the ferroptosis process.

With respect to the TME, tumors with high NOTCH2 expression exhibited increased infiltration of M2 macrophages. As a key subset of tumor-associated macrophages (TAMs), M2 macrophages are closely linked to immunosuppression and tumor progression (53). It has been reported that global activation of NOTCH signaling generally induces the M1 phenotype, while inhibition of this pathway tends to promote the M2 phenotype (54). However, studies focusing on NOTCH2 have uncovered more complex and context-specific regulatory patterns. Particularly in the TME, NOTCH2 function is more inclined to drive the formation of M2-like tumor-associated macrophages (TAMs). For example, in colorectal cancer, NOTCH2 expression was shown to enhance GATA3-mediated IL-4 secretion, directly promoting M2-type TAM polarization. This evidence confirms NOTCH2 acts as a positive regulator of M2 polarization under specific pathological conditions (55). Additionally, miR-487a derived from osteosarcoma cells can be transferred into macrophages via small extracellular vesicles (sEVs) before promoting macrophage polarization toward an M2-like phenotype. This effect is mediated by targeting NOTCH2 and activating the GATA3 pathway (56). Therefore, NOTCH2 may represent a crucial node in the M2 polarization regulatory network, yet its specific downstream signaling mechanisms in pancreatic cancer require further investigation. Consistent with these findings, patients with high NOTCH2 expression exhibited elevated TIDE and immune dysfunction scores, both of which are associated with poor responsiveness to ICIs. Overall, these findings suggest that NOTCH2 may facilitate immune evasion by fostering an immunosuppressive tumor microenvironment, ultimately contributing to tumor progression and therapeutic resistance.

Finally, results from iron staining and oxidative stress analysis revealed that knockdown of NOTCH2 was associated with iron accumulation and increased lipid peroxidation, suggesting that NOTCH2 may contribute to pancreatic cancer progression by modulating ferroptosis and redox homeostasis. Although direct evidence linking NOTCH2 to the regulation of ferroptosis is currently lacking, our analysis revealed enrichment of the KEAP-NFE2L2 signaling pathway in tumors with high NOTCH2 expression. KEAP-NFE2L2 pathway is recognized as a principal regulatory axis in the cellular response to oxidative and electrophilic stress. Studies have suggested that the ROS-Nrf2 pathway can mediate TGF-β-induced EMT and is involved in the activation of NOTCH signaling (57). Furthermore, crosstalk exists between the NRF2 and NOTCH signaling pathways in lung cancer (58). Therefore, NOTCH2 may indirectly affect cellular redox homeostasis and ferroptosis sensitivity by regulating the activity or expression of NRF2.

Given the multiple pro-cancer roles of NOTCH2 in the initiation, progression, maintenance of CSC properties, and chemoresistance of pancreatic cancer, NOTCH2 represented an attractive potential therapeutic target. Drug development targeting the NOTCH signaling pathway had mainly focused on the following directions: 1.γ-Secretase Inhibitors (GSIs): These drugs block the activation of all NOTCH receptors by inhibiting the activity of γ-secretase, making them the most extensively studied NOTCH pathway inhibitors. Several GSIs, like PF-03084014, had undergone preclinical or early-phase clinical trials in various tumors, including pancreatic cancer. However, their pan-NOTCH activity resulted in poor selectivity, simultaneously inhibiting NOTCH1 and NOTCH2, which were essential for normal tissue function (particularly in the gastrointestinal tract). This induces severe dose-limiting gastrointestinal toxicity, greatly restricting their clinical application (59, 60). 2.Monoclonal Antibodies (mAbs): To overcome the toxicity issues of GSIs, the development of monoclonal antibodies targeting specific NOTCH receptors or ligands has emerged as a more promising strategy. Several antibodies, including tarextumab and brontictuzumab, entered early-phase clinical trials. In theory, the development of highly selective anti-NOTCH2 antibodies was expected to provide therapeutic efficacy while minimizing gastrointestinal toxicity caused by NOTCH1 inhibition (61). 3.Emerging Technologies: Other approaches included small molecules or peptides that interfered with the NICD-CSL complex, RNA interference to specifically downregulate NOTCH2 expression, and Proteolysis-Targeting Chimeras (PROTACs) designed to degrade NOTCH2. However, to date, no highly selective inhibitors, antibodies, or PROTAC molecules specifically targeting NOTCH2 had progressed into clinical trials. Most clinical-stage agents remained pan-NOTCH inhibitors or were directed against other NOTCH family members (62). This indicates that there is still a long way to go from the basic research discovery of NOTCH2’s importance to the successful development of specific targeted drugs applicable in clinical practice.

Taken together, NOTCH2 plays a complex yet critical pro-tumorigenic role in the pathophysiological progression of pancreatic cancer. It not only drove the transition from precancerous lesions to invasive tumors but also regulated proliferation, invasion, stemness, and chemoresistance. These functions established NOTCH2 as a promising prognostic marker and therapeutic target. Nonetheless, major challenges persisted in validating its clinical relevance, elucidating its mechanisms in greater depth, and developing specific targeted therapeutics. Future research is urgently needed to overcome these barriers and translate NOTCH2-directed strategies into clinical applications.

## 5 Conclusions

In this study, we established a robust prognostic signature for pancreatic cancer and identified NOTCH2 as a potential prognostic biomarker. Preliminary evidence suggests that NOTCH2 may contribute to the malignant progression of pancreatic cancer by regulating ferroptosis and promoting an immunosuppressive tumor microenvironment. These findings indicate that NOTCH2-mediated ferroptosis modulation may represent a promising therapeutic target in pancreatic cancer.

## Data Availability

The original contributions presented in the study are included in the article/**Supplementary Material**. Further inquiries can be directed to the corresponding author.
