# Correction: Development of a ferroptosis-related signature and identification of NOTCH2 as a novel prognostic biomarker in pancreatic cancer

**DOI:** 10.3389/fimmu.2026.1788460

**Published:** 2026-01-23

**Authors:** Siyi Zhang, Xiaoxuan Li, Xiang-Xue Li, Zi-Heng Zhang, Kai-Hui Zhu, Jing Guo

**Affiliations:** 1 Department of Oncology, The Affiliated Hospital of Qingdao University, Qingdao, Shandong, China; 2 Department of Gastroenterology, Huangdao District People’s Hospital, Qingdao, Shandong, China

**Keywords:** ferroptosis, notch2, pancreatic cancer, immune infiltration, prognostic biomarker

In the published article, there was an error in the author list. Zhu kaihui was designated as a co-corresponding author in both the submission system and the manuscript, but this was not reflected in the final published version. Accordingly, the corrected author list appears below “Siyi Zhang^1^, Xiaoxuan Li^1^, Xiang-Xue Li^1^, Zi-Heng Zhang^1^, Kai-Hui Zhu^2*^, Jing Guo^1*^”.

